# Daclatasvir plus Sofosbuvir with or without ribavirin in patients with chronic Hepatitis C genotype 3a in Pakistani population - A real world experience

**DOI:** 10.12669/pjms.35.2.637

**Published:** 2019

**Authors:** Zamir Butt, Syed Muhammad Ali Shah

**Affiliations:** 1 *Dr. Zamir Butt, MBBS, FCPS. Department of Medicine, Aziz Bhatti Shaheed Teaching Hospital, Nawaz Sharif Medical College, Gujrat, Punjab, Pakistan*; 2 *Dr. Syed Muhammad Ali Shah, MBBS. Department of Medicine, Aziz Bhatti Shaheed Teaching Hospital, Nawaz Sharif Medical College, Gujrat, Punjab, Pakistan*

**Keywords:** Chronic hepatitis C, Daclatasvir, Genotype 3a. Sofosbuvir

## Abstract

**Background and Objectives:**

Pakistan is among leading countries of world in prevalence of chronic hepatitis C Daclatasvir plus sofosbuvir is recommended for treatment of CHC. The purpose of study was to determine the sustained virological response in patients with chronic viral hepatitis C genotype 3a irrespective of previous treatment experience or presence of liver cirrhosis.

**Methods:**

Open label observational study was conducted at ABSTH Gujrat from January 2017 to April 2018 using non-probability purposive sampling. Patients chronically infected with hepatitis C virus having genotype 3a irrespective of presence of cirrhosis or previous treatment experience were included. Treatment naive patients without cirrhosis were given 12 weeks regimen of daily daclatasvir 60mg along with daily sofosbuvir 400mg. Patients with either compensated cirrhosis or treatment experienced were given 24 weeks regimen of daily daclatasvir 60mg along with daily sofosbuvir 400mg with weight based ribavirin. Data analysis was done using SPSS 20.0

**Results:**

Total 125 patients were included in study out of which 42 (33.6%) were male and 83 (66.4%) were female. Early virological response and end treatment response was achieved by 124 (99.2%) patients. Twenty four patients were lost to further follow-up and SVR_24_ was available for 101(80.8%) patients out of which 48 were having cirrhosis and 53 were without cirrhosis. SVR_24_ was achieved by 96 patients (95%). Virological response was better in treatment naive patients and without cirrhosis compared to treatment experienced and those with cirrhosis.

**Conclusion:**

Daclatasvir plus sofosbuvir is an effective combination in patients with chronic hepatitis C genotype 3a infection.

## INTRODUCTION

Chronic hepatitis C affects 71 million people globally according to WHO estimates.^1^ Pakistan is unfortunately among the leading countries in prevalence of hepatitis C with an estimated prevalence of 6.7%^2^ and a recent study from Pakistan suggested the prevalence of HCV to be 8.64% and genotype 3a being most common in Pakistan.^3^ However one recent large scale survey conducted in Pakistan suggested the prevalence to be 4.9% which is also alarming.^4^ Chronicity of HCV infection leads to cirrhosis of liver and its complications including hepatocellular carcinoma and death of patients.^5^

Decreased prices of DAAs and advent of generics has boosted up the efforts in treatment of chronic hepatitis C in Pakistan. Pakistan along with Egypt had half of people starting DAAs for treatment of chronic hepatitis C in the world during 2016^6^ and this number is increasing with passing time.

Twelve week regimen of once daily daclatasvir plus sofosbuvir has showen good results in patients with HCV genotype 3 with sustained virological response of 91%.^7^ ALLY 3 phase III study also showed good virological response in genotype 3 treatment naive and treatment experienced patients.^8^ Treatment naive patients had 96% SVR rates as compared to treatment experienced patients (86%) with higher SVR rates in patients without cirrhosis. ALLY 3+ study also showed good SVR rates in patients with advance liver disease when combination of daclatasvir and sofosbuvir was augmented with weight based ribavirin.^9^

Combination of daclatasvir and sofosbuvir is recommended for genotype 3 patients according to AASLD 2017 guidelines having a strong evidence. Recommended duration is 12 weeks in patients without cirrhosis and 24 weeks in patients with cirrhosis when weight based ribavirin is added to the regimen irrespective of previous peglated interferon plus ribavirin treatment.^10^ However recent EASL guidelines for treatment of HCV has not included this combination in treatment of genotype 3 infection.^11^

There are no studies previously published in Pakistan regarding efficacy of daclatasvir plus sofosbuvir in patients with hepatitis C genotype 3a up to best of our knowledge. As this is an effective regimen its efficacy should be evaluated in a population having high incidence of infection. Thus the purpose of study was to determine the sustained virological response in patients with chronic viral hepatitis C genotype 3a irrespective of previous treatment experience or presence of liver cirrhosis.

## METHODS

This open label observational study was conducted at Aziz Bhatti Shaheed Teaching Hospital Gujrat from January 2017 to April 2018. Patients were included in study after informed consent and approval of ethical committee of hospital. Patients chronically infected with hepatitis C virus having genotype 3a irrespective of presence of cirrhosis or previous treatment experience with interferon plus ribavirin were included. Patients were given this regimen due to free availability of DAAs in government setups and those who could not afford the first line regimen i.e combination of Velpatasvir plus Sofosbuvir.

Presence of chronic hepatitis C was confirmed with baseline quantitative PCR testing and a value of > 15 ng/ml was considered positive. Genotype testing was done for every patient by University of Gujrat Laboratory free of cost and those with genotype 3a were selected using non-probability purposive sampling. Patients having liver cirrhosis were confirmed by abdominal ultrasound done by consultant radiologist and presence of coarse echotexture of liver was considered as liver cirrhosis. Although Shear Wave Elastography and Fibroscan are recommended but non-availability of these modalities led us to rely on ultrasound for presence of cirrhosis. Severity of liver disease was assessed using Child Pugh Score and patients with score 5-6 were defined as Child Class A, 7-9 as Child Class B and 10-15 as Child Class C. Patients with Child class A and B were included while those having Child class C were excluded from study.

Treatment experienced patients were further divided in two groups. Patients who did not respond to 24 weeks interferon plus ribavirin were classified as non-responders while those who had a positive PCR after achieving ETR with 24 weeks interferon plus ribavirin treatment were classified as relapsers.

Treatment naive patients without cirrhosis were given 12 weeks regimen of daily daclatasvir 60mg along with daily sofosbuvir 400mg. Patients with either compensated cirrhosis (Child Class A & B) or treatment experienced were given 24 weeks regimen of daily daclatasvir 60mg along with daily sofosbuvir 400mg with weight based ribavirin. PCR was done to assess the treatment response at four weeks of treatment (Early Virological Response or EVR), at end of 12 or 24 weeks treatment (End Treatment Response or ETR). Primary end point of study was to determine sustained virological response (SVR_24_) 24 weeks after completion of treatment.

Data analysis was done using SPSS 20.0. Continuous variables like age were expressed as mean + SD while categorical variables such as SVR, ETR or EVR were expressed as percentage.

## RESULTS

One hundred twenty five patients were included in study out of which 42 (33.6%) were male and 83 (66.4%) were female. Mean age was 47.06 + 10.8 years. 102 (81.6%) patients were treatment naive, 7 (5.6%) treatment non-responders and 16 (12.8%) were relapsers. Fifty eight (46.4%) patients had cirrhosis including 43 treatment naive, 4 non-responders and 11 relapsers patients. Fifty patients with cirrhosis had Child Class A while 8 patients had Child Class B.

Early virological response (EVR) and end treatment response (ETR) was achieved by 123(98.4%) and 124 (99.2%) patients respectively. Taking in account treatment history; treatment naive, relapsers and non-responders achieved EVR of 99%, 100% and 85.7% and ETR was achieved by 99%, 100% and 100% respectively. One fifth of patients (24) were lost to further follow-up who were alive and did not report back despite multiple reminders. SVR_24_ was available for 101(80.8%) patients out of which 48 were having cirrhosis and 53 were without cirrhosis. SVR_24_ was achieved by 96(95%) patients out of 101. Patients who did not achieve SVR_24_ included both treatment naive and experienced patients and patients with and without cirrhosis. SVR_24_ in patients with cirrhosis were 91.66% while in those without cirrhosis were 98.11%. 97.65% treatment naive patients achieved SVR_24_ however only 90.9% relapsers and 60% non-responders achieved SVR_24_. Results are depicted in Table-I.

**Table-I:** Treatment Response after Daclatasvir plus Sofosbuvir with or without Ribavirin.

	EVR	ETR	SVR_24_
Total	123/125 (98.4%)	124/125 (99.2%)	96/101 (95%)
***Gender***			
Male	41/42 (97.6%)	41/42 (97.6%)	32/33 (96.97%)
Female	82/83 (98.8%)	83/83 (100%)	64/68 (94.12%)
***Treatment History***			
Treatment Naive	101/102 (99%)	101/102 (99%)	83/85 (97.65%)
Non-Responders	6/7 (85.7%)	7/7 (100%)	3/5 (60%)
Relapsers	16/16 (100%)	16/16 (100%)	10/11 (90.9%)
***Cirrhosis***			
Present	57/58 (98.3%)	57/58 (98.3%)	44/48 (91.66%)
Absent	68/67 (98.5%)	67/67 (100%)	52/53 (98.11%)
***Child Pugh Class***			
Class A	49/50 (98%)	49/50 (98%)	38/41 (92.7%)
Class B	8/8 (100%)	8/8 (100%)	6/7 (85.7%)

***Abbreviations:*** EVR - Early Virological Response, ETR - End Treatment Response,

SVR_24_ - Sustained Virological Response at 24 weeks.

## DISCUSSION

This study shows that combination of daily daclatasvir plus daily sofosbuvir with or without weight based ribavirin is highly effective in Pakistani population with HCV genotype 3a and independent of presence of cirrhosis or previous treatment with interferon plus weight based ribavirin. SVR_24_ rates were more in patients without cirrhosis than as compared to cirrhosis and treatment naive patients as compared to treatment experienced patients. However no statistical significance of cirrhosis and treatment history were found.

Results of this study are comparable to ALLY 3 phase III study by Nelson et al.^8^ Treatment response in treatment experienced and treatment naive patients in this study were 100% and 99% at week four (EVR) and end of treatment (ETR) respectively. These are similar results to ALLY-3 study in which 94% treatment naive and 98% treatment experienced patients showed undetectable HCV RNA at week four of treatment and 99% treatment naive and 100% treatment experienced patients resulted in undetectable viral RNA. SVR12 in ALLY 3 study were 96% which are comparable to results in this study. Nelson et al. showed that SVR 12 were higher in treatment naive patients as compared to treatment experienced and in those without cirrhosis as compared to those with cirrhosis which further strengthen the results of this study^8^. However they considered genotype 3 as compared to genotype 3a in this study.

Patients with compensated cirrhosis has good treatment response in this study. 98.3% patients with compensated cirrhosis achieved EVR and ETR. ALLY 3+ study also determined the role of daclatasvir and sofosbuvir in patients with compensated cirrhosis and resulting in 83.3% patients achieving treatment response at week 4 and 100% at end of treatment.^9^ SVR12 rate in patients with cirrhosis in ALLY 3+ were 83% with 12 week regimen compared to 91.66% in this study. Difference in results may be due to different sample sizes (almost double in this study) and different treatment durations.

Welzel et al. conducted a study about efficacy of daclatasvir plus sofosbuvir with or without ribavirin in HCV patients. HCV RNA was undetectable in 73% patients at week 4, 97% at week 12 and >99% at week 24. However they included all genotypes of HCV. In genotype 3, 92% patients achieved SVR which was minimally less in treatment experienced patients having decompensated cirrhosis.^12^ Their findings suggest results similar to this study. Patients with cirrhosis and treatment experience had low SVR_24_ rates as compared to treatment naive and without cirrhosis. Alonso et al. also found a high SVR rates (94%) among patients with HCV genotype 3 infection treated with sofosbuvir plus daclatasvir^13^ which are comparable to results of this study (SVR_24_ = 95%).

Mehta et al. conducted a study in HCV genotype 3 patients in India using combination of sofosbuvir plus daclatasvir and 97.3% patients achieved SVR showing it to be highly effective regimen in genotype 3^14^ which also supports the result of this study.

Study by Ferrieria et al. showed lower SVR rates (84.7%) in patients with genotype 3 taking sofosbuvir plus daclatasvir regimen which do not augment results of this study. However they found out no significant association of presence of cirrhosis or treatment experience with achieving SVR which are consistent with results of this study.^15^

This is perhaps first study in Pakistan determining the role sofosbuvir plus daclatasvir in treatment of chronic hepatitis C genotype 3a infection. Although SVR at 12 week is considered the end point in many studies, SVR at 24 weeks was focused in this study. However 19.2% patients lost to follow up which shows a need for a free of cost screening, treatment and follow up program for treatment of hepatitis C at national level owing to high prevalence of chronic hepatitis C Pakistan.^2^-^4^ This study was done on a small sample size, further large scale studies should be conducted to get the exact picture of efficacy of daclatasvir and sofosbuvir combination.

Study showed good outcomes in treatment of hepatitis C genotype 3a which is prevalent genotype in Pakistan.^3^ Although current EASL guidelines do not recommend the use of this combination^11^, the availability of only few DAAs in Pakistan makes it an affordable and effective option. Further large scale studies are needed at national level regarding different treatment options being used in Pakistani population.

## CONCLUSION

Daclatasvir plus sofosbuvir is a highly effective combination in patients with chronic hepatitis C genotype 3a infection in Pakistani population and independent of treatment history and presence of cirrhosis.

### Authors’ Contribution

**ZB:** Conceived the study, collected data and did final review and approval of manuscript.

**SMAS:** Planned the study, did statistical analysis and wrote manuscript.
